# Difficult clinical management of antituberculosis DRESS syndrome complicated by MRSA infection: A case report

**DOI:** 10.1097/MD.0000000000006346

**Published:** 2017-03-24

**Authors:** Li Wang, Lin-Feng Li

**Affiliations:** Department of Dermatology, Allergy and Clinical Immunology Centre, Beijing Friendship Hospital, Capital Medical University, Beijing, China.

**Keywords:** antituberculosis, case report, drug hypersensitivity syndrome, drug reaction, drug reaction with eosinophilia and systemic symptoms, methicillin-resistant *Staphylococcus aureus*

## Abstract

**Rationale:**

Drug reaction with eosinophilia and systemic symptoms (DRESS) syndrome is a severe drug-induced hypersensitivity reaction characterized by skin rash, fever, blood abnormalities, and multiple organ involvement. The diagnosis of DRESS syndrome is often delayed because of its variable presentation. Prompt withdrawal of the culprit drug is the definitive treatment. DRESS syndrome induced by antituberculosis drugs has rarely been reported.

**Patient Concerns:**

A 50-year-old man admitted to our hospital with recurrent episodes of progressive rash, fever, eosinophilia, lymphadenopathy, hepatic, and pulmonary involvement were experienced after repeat trials of the same antituberculosis drugs.

**Diagnoses:**

We diagnosed it as DRESS caused by antituberculosis drugs.

**Interventions:**

The case responded well to treatment with systemic corticosteroids and intravenous immunoglobulins. However, repeated bouts of infection with methicillin-resistant *Staphylococcus aureus* occurred during treatment (clavicular osteomyelitis and knee septic arthritis). He was cured after treatment with linezolid.

**Outcomes:**

The patient was discharged on day 112. At 8-month follow-up, there was no relapse of drug eruption and joint swelling.

**Lessons:**

Early diagnosis and prompt withdrawal of all suspected drugs is a key tenet of the treatment of DRESS. Our case report highlights the risks inherent in delayed diagnosis of DRESS and the challenges in the clinical management of this condition. Pulmonary manifestations with radiological changes on chest X-ray and CT can be seen in DRESS. These changes need to be differentiated from those caused by pulmonary infections. Clavicular osteomyelitis infected with MRSA may be caused by iatrogenic injury during subclavian vein catheterization. This type of MRSA infections should be treated for 4 to 6 weeks. Blood eosinophilia could be a useful marker of disease progression and treatment response in patients with DRESS. However, more experience and clinical evidence is needed to confirm this.

## 1 Introduction

Drug reaction with eosinophilia and systemic symptoms (DRESS) syndrome was named by Bocquet in 1996.^[1]^ The clinical manifestations of DRESS include drug-induced general skin rash, fever, lymphadenopathy, abnormal hematological findings, liver damage, as well as damage to other internal organs such as kidney, lung, heart, and pancreas. The condition is potentially life-threatening.^[2]^ The most common drugs associated with DRESS include anticonvulsants (mostly aromatase derivatives), antimicrobial agents, particularly penicillin and sulfonamide-based agents, and antipyretic/antiinflammatory analgesics.^[3]^ DRESS has a relatively long latency period (between 2 and 8 weeks), and symptoms may persist for up to 2 weeks after the discontinuation of the culprit drug. Mortality rate is approximately 10%.^[4]^ Early diagnosis and immediate withdrawal of the suspected drug is a key tenet of management of DRESS.^[5]^ The patient had probably developed a hypersensitivity reaction to antituberculosis drugs. He also developed recurrent bouts of clavicular osteomyelitis, sepsis, and knee septic arthritis caused by hospital-acquired infection with methicillin-resistant *Staphylococcus aureus* (MRSA). We summarize our experience with the diagnosis and treatment of this case of DRESS syndrome.

## 2 Case presentation

On June 12, 2015, a 50-year-old man presented at the emergency department with fever and diffuse erythema over the entire body. Six months back, he was diagnosed with pulmonary tuberculosis and tuberculous pleurisy at a tuberculosis clinic, and was started on antitubercular therapy (isoniazid [0.3 g/qd], rifampicin [0.45 g/qd], and pyrazinamide [0.5 g/q12 h]). Sixteen days after the initiation of treatment, he developed pruritic erythema and papules over his abdomen and lower extremities. The skin lesions gradually resolved after withdrawal of antituberculosis drugs. One month prior to presentation, he again developed skin eruptions after a repeat trial of the same antituberculosis drugs for 14 days. He had a history of gastric perforation repair 1 year ago.

**Figure 1:**
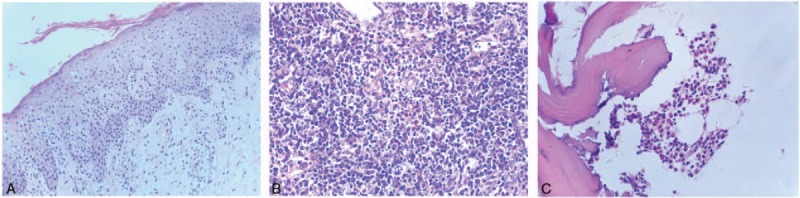
(A) Histological examination of skin eruption shows intraepidermal spongiosis, perivascular lymphocytic infiltration, and presence of a few eosinophils in the dermis; hematoxylin and eosin (H&E), ×200. (B) Histological examination of inguinal lymph node showing benign hyperplasia with eosinophilia; H&E, ×400. (C) Histological examination of bone marrow showing hypercellular marrow with a high percentage of eosinophils (up to 33%); H&E, ×400.

At admission, generalized papules and erythema were observed over his face, trunk, extremities, palms, and soles. The patient's weight was 66 kg, body temperature was 39.4 °C, blood pressure was 110/60 mm Hg, and heart rate was 96 beats per minute. Blood routine examination showed leukocytosis with a white blood cell count of 14.88 × 10^9^/L (72.2% [10.74 × 10^9^ /L] neutrophils and 19.3% [2.87 × 10^9^ /L] eosinophils). No atypical lymphocytes were detected on peripheral blood smear. Blood biochemistry test results were notable for alanine aminotransferase 85 U/L, aspartate aminotransaminase 44 U/L, lactate dehydrogenase 853 U/L, and albumin 29.1 g/L. Renal function and common tumor markers were within normal range. Urine routine examination was normal and stool occult blood test was negative. Serological tests for common viral infections and autoantibodies were negative. Blood culture was negative. Myocardial zymogram and electrocardiogram were also normal. Radiological workup revealed several new lesions when compared with his preantitubercular treatment radiological record. Chest X-ray showed new bilateral nodular opacities in both lower lung lobes. Nonenhanced chest, abdominal, and pelvic computed tomography (CT) showed bilateral ground-glass opacities and increased pulmonary effusion in the right chest; a small amount of pericardial effusion; multiple enlarged lymph nodes in the mediastinal and axillary region; and multiple enlarged intraabdominal lymph nodes. The preliminary impression was “most likely pulmonary inflammation.” After admission, his antituberculosis therapy was not stopped. Over the next 7 days, his skin lesion continued to worsen. Repeat chest X-ray examination showed no response to treatment with moxifloxacin. Histological examination of skin biopsy (Fig. 1A), inguinal lymph node needle biopsy (Fig. 1B), and bone marrow puncture and biopsy (Fig. 1C) were all compatible with DRESS syndrome. Based on the clinical, laboratory, and pathological findings, he was diagnosed as a case of DRESS syndrome; the pulmonary inflammation was probably attributable to organ involvement of DRESS. Rifampicin and pyrazinamide were withdrawn while isoniazid therapy was continued.

**Figure 2:**
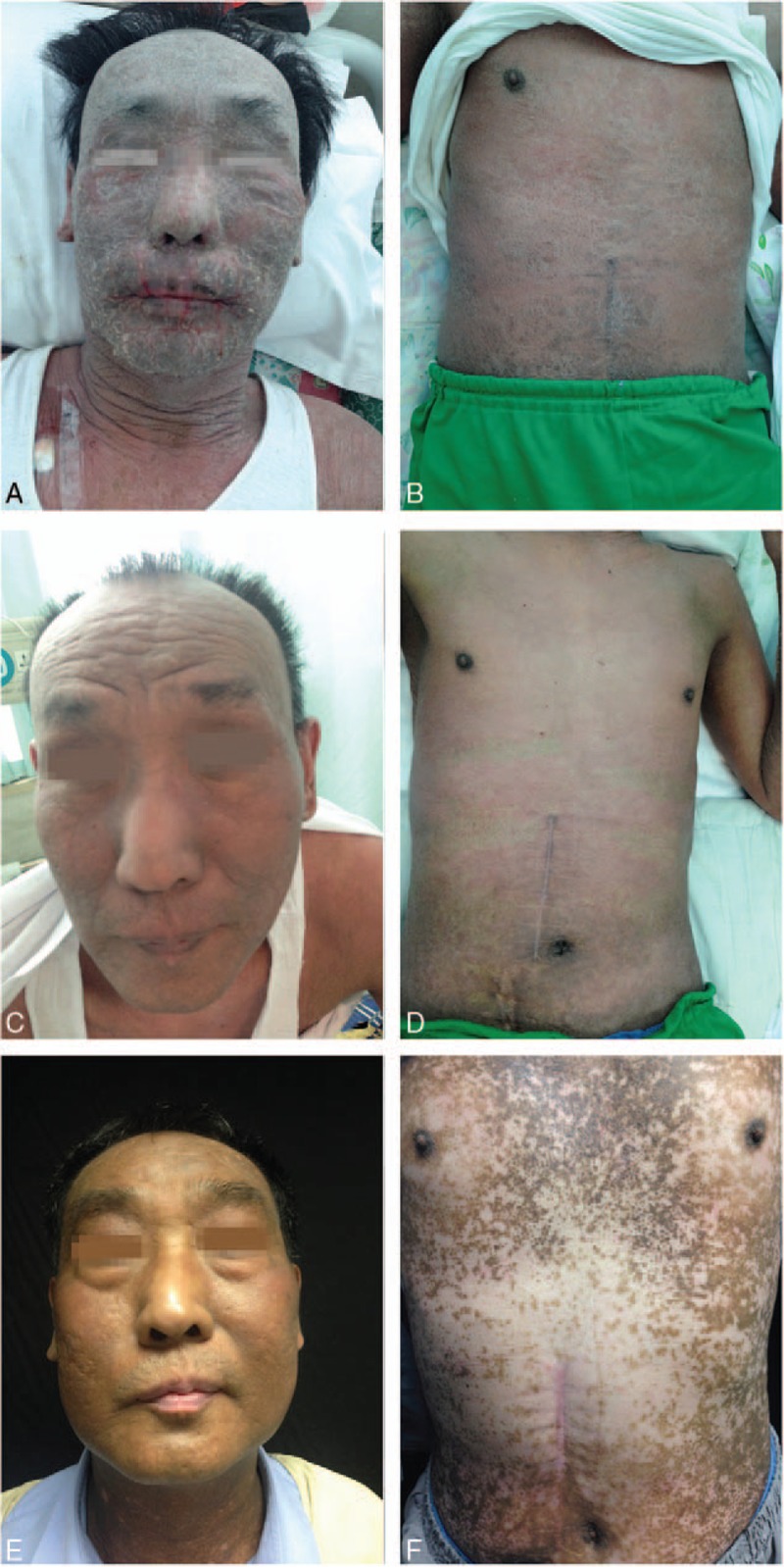
Before systematic steroids, diffuse erythema, swelling and scales on the face (A) and trunk (B). After systematic steroids, mild erythema on the face (C) and trunk (D). 5-month follow-up, extensive hyperpigmentation on the face (E) and trunk (F).

Owing to swollen upper extremities, right subclavian vein catheterization was performed for peripheral venous access. Several days later (day 19), he developed generalized erythema and swelling over the whole body with bran-like scales (Fig. 2A, B). After dermatological consultation, DRESS was possibly considered to be induced by isoniazid, isoniazid was discontinued, and oral prednisone (60 mg/day) therapy was initiated. Subsequently, his skin lesions improved, temperature as well as liver enzymes normalized, and the percentage of blood eosinophils decreased to 11.6% (0.87 × 10^9^/L).

On day 26, the patient complained of swelling in the right sternoclavicular joint region and developed fever up to 39 °C. A new chest X-ray showed decreased nodular opacities. MRSA was isolated on blood culture. Based on antibiotic sensitivity tests, intravenous linezolid (1.2 g/day) was administered for 14 days. Considering the adverse effects of high-dose steroid therapy in a patient with MRSA and tuberculosis infection, his prednisone dose was reduced to 40 mg/day. Although the soft tissue swelling in the sternoclavicular joint subsided and the venous catheter removed on day 41, he developed erythroderma over the whole body and a resurgence in blood eosinophil counts was observed (3.92 × 10^9^/L). Consequently, prednisone was replaced by intravenous methylprednisolone (80 mg/day), and intravenous immunoglobulin (20 g/day) was administered for 5 days. The patient's skin lesion improved rapidly. On day 50, his blood eosinophil count had decreased to 0.61 × 10^9^ /L with complete resolution of the scales and swelling (Fig. 2C, D). Following this, the dose of steroids was gradually reduced.

**Figure 3:**
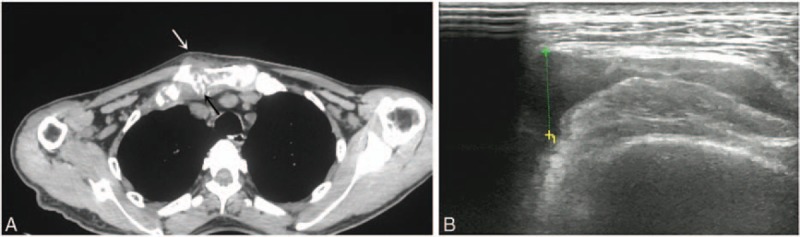
(A) Computed tomography (CT) radiograph showing bone destruction (black arrow) and soft tissue swelling (white arrow) in the right sternoclavicular joint. (B) Ultrasonogram showing effusion in the knee joints.

On day 61, the patient again developed fever and complained of swelling and pain in the right sternoclavicular and bilateral knee joints. CT scan of right sternoclavicular joint was consistent with acute osteomyelitis (Fig. 3A). Ultrasonography showed effusion in the knee joints (Fig. 3B). MRSA was isolated from blood and pus from the swollen joints. He was immediately commenced on intravenous linezolid (1.2 g/day) again and his temperature rapidly returned to normal 1 day later. The antibiotic treatment was maintained for 1 month until swelling in all joints was resolved. Over the next several days, the systematic steroids were tapered.

The patient was discharged on day 112. One month later, his skin eruption was replaced by hyperpigmentation completely. At 5-month follow-up, he was weaned off systemic steroids with fading skin pigmentation (Fig. 2E, F). At 8-month follow-up, there was no relapse of drug eruption and joint swelling. He has been on antituberculosis drugs (streptomycin and moxifloxacin) for 2 months without any associated complications. The patient signed an informed consent statement for publication, and the study was a retrospective analysis of clinical case, so ethical approval to report this case was not required.

## 3 Discussion

DRESS syndrome is typically characterized by a series of severe systemic complications with diverse manifestations, which can pose a considerable diagnostic and therapeutic challenge.^[4,6,7]^ Our patient qualified 6 out of the 7 RegiSCAR criteria at admission.^[7,8]^

In addition to meeting the criteria, DRESS syndrome is a diagnosis of exclusion. Severe bacterial and viral infections, malignancies, and autoimmune diseases should be ruled out before arriving at a definitive diagnosis.^[9]^ Our patient presented radiological signs of pulmonary infection with increased nodular opacities on chest X-ray as well as bilateral ground-glass opacities and increased pleural effusion in the right chest on nonenhanced chest CT. Given the lack of any obvious regression in lung inflammation after 1-week treatment with moxifloxacin, and the results of other auxiliary examinations, the diagnosis was revised to DRESS syndrome. His pulmonary inflammation was probably attributable to organ involvement of DRESS. DRESS may be associated with pulmonary manifestations which may manifest as changes in chest X-ray and CT. This should be differentiated from pulmonary infection.^[10]^ The patient did not respond to moxifloxacin, but systemic steroid treatment that was started on day-19 led to a decrease in opacities by day-26. This also suggests that the observed pulmonary lesions were a manifestation of DRESS. The choice of treatment was initially misled by the radiological findings. This was one of the reasons for the delayed diagnosis in this patient.

Antitubercular drugs that are known to cause DRESS include isoniazid, rifampicin, streptomycin, and pyrazinamide.^[11,12]^ Our patient developed mild symptoms of drug eruption after initiation of antituberculosis therapy and presented with typical symptoms of DRESS which started 2 weeks after reinitiating of treatment with the same antituberculosis drugs. Therefore, the causative drugs were probably one or more of the 3 antituberculosis drugs (isoniazid, rifampicin, and pyrazinamide).

In view of his serious condition, drug challenge test with the 3 antitubercular drugs was not conducted.^[13]^ Even in the absence of rifampicin and pyrazinamide, the patient's DRESS symptoms aggravated gradually with administration of isoniazid. As the severity of drug hypersensitivity is associated with the duration of exposure to the offending drugs after the onset of symptoms,^[14]^ we speculated the most probable causative drug to be isoniazid. The initial worsening of the patient's condition was induced by failure to withdraw the offending drugs timely. The patient's initial treatment at the tuberculosis clinic (isoniazid, rifampicin, and pyrazinamide) caused a mild skin rash, which resolved after the discontinuation of those. When the patient sought medical care at the tuberculosis clinic again, the same drugs were prescribed because the treating physicians probably underestimated the severity of the adverse reactions that can be caused by reintroduction of the same drugs. The patient was later treated at the emergency department for adverse drug reaction. The emergency physician withdrew rifampicin and pyrazinamide, and the patient was administered oral antihistamines and local steroid treatment. However, owing to continued aggravation of the rash, dermatological consultation was sought. Isoniazid was discontinued for the first time only after the advice of dermatologist. This suggests that the emergency physicians were probably not well-aware of the potential life-threatening complications associated with allergy to antituberculosis drugs. Therefore, a dermatologist should be consulted on suspicion of a severe adverse drug reaction to avoid delayed diagnosis. So far, the only undisputed way to treat DRESS is prompt withdrawal of the offending drug, and no consensus guidelines exist for its management.^[15]^ The current therapeutic strategies are empirical. Systemic corticosteroid therapy at 1 to 2 mg/kg/day for an average of 6 weeks was reported to be effective for multiple organ involvement.^[12,16]^ In the present case, considering the possible adverse effects on his pulmonary tuberculosis and gastrointestinal tract, systemic steroids were not administered on admission, which is probably the 2nd cause of the worsening of his condition. Moreover, prednisone was initially administered at the dose of 60 mg/day (<1 mg/kg/day) and later rapidly reduced to 40 mg/day rapidly after detection of MRSA infection, which may have been the 3rd cause of deterioration of DRESS.^[17]^

To our knowledge, assessing the severity of DRESS remains an issue.^[15]^ In our patient, the blood eosinophil counts showed a positive correlation with the severity of DRESS symptoms. After systemic steroid treatment for 10 days, the patient's blood eosinophil count decreased from 2.87 to 0.87 × 10^9^/L, and his body temperature and liver enzymes normalized. On rapid reduction in steroid dose to 40 mg/day, a resurgence in eosinophil counts (3.92 × 10^9^ /L) was observed within a week, which was accompanied by aggravation of skin rash. The changes in blood eosinophil counts paralleled the severity of skin rash and internal organ damage. The present case suggests that blood eosinophilia could be a useful marker for disease progression and treatment response in patients with DRESS. Blood eosinophilia preceded to the aggravation in skin rash and could therefore be an early sign of disease progression. However, more cases and more clinical evidence are needed to confirm this. Clavicular osteomyelitis is a rare clinical entity and can occur as a complication of subclavian vein catheterization and neck surgery.^[18]^ MRSA is known to be endemic in hospital settings across the world and accounts for 40% to 60% of all nosocomial *Staphylococcus aureus* infections. Additionally, systemic causes such as use of corticosteroids, intravenous drug abuse, tuberculosis, diabetes, and cancers, can predispose to infectious complications.^[19]^ We speculate that our patient acquired MRSA essentially due to iatrogenic injury following right subclavian vein catheterization. Subsequently, hematogenous dissemination of infection led to MRSA-associated clavicular osteomyelitis and septic arthritis at the knee joint.

The patient experienced a relapse in MRSA infection in hospital. Infectious Diseases Society of America recommends 4 to 6 week treatment for MRSA infection in seriously ill patients.^[20]^ We believe that the recurrence of MRSA infection was probably due to insufficient duration of treatment.

## 4 Conclusion

Our case report highlights the risk inherent in delayed diagnosis of DRESS and challenges in clinical management of this condition. The key to the treatment of DRESS is early diagnosis and immediate withdrawal of all suspected drugs. When an adverse drug reaction is observed, reuse of the same drug could cause more severe adverse drug reactions the 2nd time. When severe adverse drug reactions are suspected, a dermatologist should be consulted and the suspected drug should be discontinued immediately. Delayed diagnosis could be life-threatening. Pulmonary manifestations with radiological changes on chest X-ray and CT can be seen in DRESS. These changes need to be differentiated from those caused by pulmonary infection. Systemic steroid treatment is effective against DRESS. If the patient suffers from a condition that makes the use of steroids inappropriate, intravenous immunoglobulin might be a possible alternative. Decrease in the steroid dose too quickly can lead to rebound symptoms. Clavicular osteomyelitis infected with MRSA might be caused by iatrogenic injury during right subclavian vein catheterization, which may lead to septicemia and hematogenous dissemination of MRSA causing joint infections. This type of MRSA infections should be treated for 4 to 6 weeks. Blood eosinophilia could be a useful marker of disease progression and treatment response in patients with DRESS. However, more experience and clinical evidence is needed to confirm this.
